# Measuring Human-Animal Attachment in a Large U.S. Survey: Two Brief Measures for Children and Their Primary Caregivers

**DOI:** 10.3389/fpubh.2019.00107

**Published:** 2019-05-14

**Authors:** Regina M. Bures, Megan Kiely Mueller, Nancy R. Gee

**Affiliations:** ^1^Eunice Kennedy Shriver National Institute of Child Health and Human Development (NICHD), Bethesda, MD, United States; ^2^Cummings School of Veterinary Medicine, Tufts University, North Grafton, MA, United States; ^3^Tisch College of Civic Life, Tufts University, Medford, MA, United States; ^4^Department of Psychology, SUNY at Fredonia, Fredonia, NY, United States; ^5^WALTHAM Centre for Pet Nutrition, Melton Mowbray, United Kingdom

**Keywords:** child development, human-animal interaction (HAI), population representative sample, measurement, Panel Study of Income Dynamics (PSID)

## Abstract

Researchers in the human-animal interaction (HAI) field face a challenge in generalizing the impact of pet ownership and companion animal interaction from small samples to larger populations. While researchers in Europe and Australia have included measures of pet ownership and attachment in surveys for some time (e.g., the Avon Longitudinal Study of Parents and Children), survey researchers in the United States have been slow to incorporate questions related to HAI in population representative studies. One reason for this may be that many of the current HAI-related measures involve long, complex scales. From the survey administration perspective, using complex scales is costly in terms of both time and money. The development and validation of brief measures of HAI will facilitate the inclusion of these measures in larger surveys. This paper describes the psychometric properties of two brief attachment measures used in the first population-representative study of child development in the United States that includes HAI items, the 2014 Panel Study of Income Dynamics (PSID) Child Development Supplement (CDS). We use two measures derived from the 29 item CENSHARE Pet Attachment Survey, one for children aged 8–17 (6-items) and one for the primary caregiver (3 items). The results suggest that such brief measures of attachment to pets are psychometrically valid and are a practical method of measuring HAI attachment in larger surveys using only a few survey items. We encourage HAI researchers to work with other ongoing surveys to incorporate these and comparable HAI measures.

## Introduction

Research on human-animal interaction (HAI) has focused on how companion animals affect the health and well-being for people of all ages [see (1–3)]. Pets are often given the status of family members (4, 5), and can play important roles in children's lives (6). The “pet effect” is that living with an animal can improve human health and well-being (7), and there is a growing body of evidence—both consistent, and inconsistent—exploring this concept. Some research has shown positive effects of HAI, including that pets can serve as a source of emotional support, ease anxiety, and encourage exercise (8). However, there are also a number of studies that have demonstrated mixed or null findings regarding the health benefits of pet ownership (2, 9). Most of the studies to date that have examined the association between companion animals and children's health, development and well-being have been based on small, non-representative sample sizes, which limits the generalizability of the findings. There remain significant gaps in our knowledge of the social and health consequences of human-animal interaction, particularly for children and child development.

Researchers investigating the impact of animals on human health and well-being recognize the importance of understanding the nature of the bond that humans and animals share [e.g., (10)]. Many researchers argue that deriving benefits from human-animal interaction is likely related to the type and depth of emotional connection between the human and the animal (8) and measures of pet attachment have been developed to assess this connection.

## Measuring Attachment to Pets

Wilson et al. (11) concluded that few measures of pet attachment existed that were reliable and valid, but, since then, several measures have been developed. Anderson (12) provided the first compendium of measures of pet attachment and other aspects of the human-animal bond. Gee and Schulenberg (13) integrated information from this compendium and others (14, 15) into recommendations focused on examining the impact of animals in educational settings. The frequent use of attachment measures within the HAI field demonstrates the importance of understanding the quality of human-animal relationships as a component of the theoretical framework for understanding HAI in families. While progress has been made in measuring HAI, the field remains focused on small studies and there remains a need for brief measures that can be incorporated into population representative surveys.

## Integration of HAI Measures into National Surveys

Several large European and Australian surveys have incorporated HAI measures but, to date, despite the fact that 68% of American households report owning at least one pet (16), few U.S. population-representative data collections do so. When HAI questions are included in U.S. surveys they often relate to a single topic such as dog ownership or dog walking (17, 18). One strategy for developing generalizable findings about HAI and child health and development is to add HAI measures into existing large scale, national surveys, such as longitudinal panel studies. This approach allows researchers to leverage robust and diverse samples and to use the longitudinal data to analyze how pet ownership affects human health and development. HAI measures can also be included cross-sectionally to allow for retrospective analyses using other measures of mental and physical health embedded within the study.

Few large-scale, longitudinal U.S. studies exist because of the extensive resources needed to develop and maintain such projects over multiple periods of measurement. The trade-offs between survey length and costs limits the addition of new measures and, as a result, pet-related questions are usually limited to dog or general pet ownership. Based on small studies, we know that HAI appears to be a critical factor in promoting human health and healthy development, especially among children. We argue that, to assess the effects of pet ownership on health and development at the population level, measures of HAI included in large studies need to go beyond simple pet ownership to include measures of the quality of the human-animal relationship. Inclusion of HAI measures in large, population-representative studies requires the construction of short-form measures of HAI attachment. This paper describes two brief measures of HAI attachment (assessing child and parent attachment to pets) that can be incorporated into larger studies and, in doing so, addresses the need for validated, short form measures of attachment that can feasibly be included in large, population representative surveys.

## Present Study

To address the need for validated, short form measures of HAI in youth, we assessed the psychometric properties of a shortened version of an existing attachment measure CENSHARE Pet Attachment Survey (PAS); (19) within a longitudinal, nationally representative study. We also assessed a similar attachment measure for the primary caregiver, typically a parent.

## Data and Methods

The Panel Study of Income Dynamics (PSID) is a longitudinal, nationally representative household survey that began in 1968. The original sample comprised over 18,000 individuals living in 5,000 families in the United States. The PSID Child Development Supplement (CDS) is a supplemental study to the main study. The first CDS study collected data on a sample of children from PSID families who were 0 to 12 years old in 1997, and followed those children over three waves, ending in 2007-08. The CDS-2014 includes all eligible children in PSID households born since 1997. This paper uses publicly available, de-identified data from the PSID CDS dataset. For additional information on the PSID CDS see: https://psidonline.isr.umich.edu/Studies.aspx.

The CDS-2014 collected data on children from the household primary caregiver (PCG) and, for older children, the children themselves. Primary caregivers are parents/guardians, typically mothers, who co-reside with CDS children and answer questions about each CDS child and about themselves and the household environment. Pet-related questions were added to the instruments for both the PCG and the older children. The questions on pet ownership and attachment were added to the PSID-CDS with funding support provided by MARS/WALTHAM™through the NICHD-MARS/WALTHAM™ public-private partnership. The inclusion of these questions in the CDS will provide baseline measures of levels of pet interaction and levels of child development that may potentially be revisited in future waves of data collection.

The older children, ages 8–17, were asked questions about the characteristics of their pets and interactions with family pets, including whether the child has a pet as well as a favorite pet, type of pet, and six questions about pet attachment. For the PCGs, questions included the number and types of pets in families and the PCG's interaction with and attitudes about their pets. The pet-related items for PCGs included number and type of current pets, whether the family had a pet 5 years ago, reasons for not owning a pet, and three questions related to pet attachment.

The attachment items for both child and parental pet attachment are based on a subset of items from the CENSHARE PAS, which was comprised of 29 items [see (12, 19)]. The items included in the CDS were chosen to address several specific aspects of pet relationships that have been hypothesized as theoretically important aspects of the human-animal bond: physical activity engagement, emotional and social support, and proximity (6, 10, 20). The response options for the CDS attachment questions (e.g., “… How often do you spend time each day playing with or exercising your pet?”) were “Almost always, often, sometimes, or never.” For the analyses, the coding of the responses was reversed so that “almost always” was coded as 4; “never” as 1. An attachment score was calculated by averaging the 6 items for the children and the 3 items for the PCGs.

In addition to the pet attachment measures, we examined several demographic and family characteristics including sex, age, family size, presence of only one child under 18 in the household, only one pet in the household, dog ownership, and cat ownership. Sex is coded female = 1, male = 0. Age and family size are continuous measures. All other measures are dichotomous: the child is the only child under 18 in the household (or the PCG reports only one child under 18 in the household), child or PCG reports a single pet (1 = one pet, 0 = more than one pet). All children and PCGs in the analyses have at least one pet. Thus, we include measures of dog ownership [has dog = 1; other pet(s) = 0] and cat ownership [has cat = 1; other pet(s) = 0]. For the children, a single pet, typically a dog or cat, is reported on; the PCGs are asked about all family pets so they may potentially report both a dog and a cat.

The PSID CDS 2014 collected data from 2,525 PCGs, typically parents, and 1,508 older children. Because the focus of this paper is the pet attachment questions, we exclude cases with missing responses to these questions. Our analytic sample includes respondents who reported having one or more pets and responded to all of the pet attachment questions (1,536 PCGs and 931 children). Principal factor analyses, correlation matrices, and additional descriptive statistics are reported in the results. Statistical analyses were conducted separately for the child and PCG samples using SAS 9.4. All results are unweighted.

## Results

Table 1 summarizes the pet attachment questions included in the PSID CDS 2014. The response distributions of the questions demonstrate distribution across the response options. The only question to which a majority of both child (nearly 81%) and PCGs (70 %) responded “Almost always” was “…how often do you consider your pet to be a member of your family?” This is also reflected in the mean for this measure (range 1 = never to 4 = almost always): for children the mean response was 3.71 (*SD* = 0.65) and for PCGs 3.49 (*SD* = 0.88).

**Table 1:** Pet Attachment Questions Included in the PSID CDS.

	**Response distributions (100%)**						
	**Never**	**Sometimes**	**Often**	**Almost always**	**Mean (SD)**	**Correlation with total**	**Factor 1**
**Child Sample (*****n*** **=** **931)**							
How often do you spend time each day playing with or exercising your pet?	5.4	29.3	37.4	27.9	2.87 (0.88)	0.5448	0.6274
How often is your pet aware of your different moods?	12.8	31.8	31.3	24.1	2.67 (0.98)	0.4365	0.5113
When you come home, how often is your pet the first one you greet?	7.5	22.4	22.2	47.9	3.11 (0.99)	0.5051	0.5834
When you feel bad, how often do you seek your pet for comfort?	15.6	33.1	24	27.3	2.63 (1.04)	0.5377	0.6168
How often do you consider your pet to be a member of your family?	1.8	5.7	11.8	80.7	3.71 (0.65)	0.4143	0.4815
How often do you have your pet near you when you study, read, or watch TV?	18.4	31	25.1	25.5	2.58 (1.06)	0.5066	0.5830
Total reliability (Cronbach Alpha)						0.7518	
Eigen value							1.95
**Primary Caregiver Sample (*****n*** **=** **1,536)**							
Do you spend time each day playing with or exercising your pet?	11.5	32.3	28.5	27.7	2.72 (0.99)	0.5921	0.6908
When you feel bad, do you seek your pet for comfort?	27.7	36.5	21.7	14.1	2.22 (1.00)	0.5722	0.6723
How often do you consider your pet to be a member of your family?	5.5	9.6	15	69.9	3.49 (0.88)	0.5050	0.5915
Total reliability (Cronbach Alpha)						0.7329	
Eigen value							1.28

Our analyses of the pet attachment questions and attachment scales follow the initial Holcomb et al. (19) approach. To examine the internal consistency of the two sets of pet attachment measures, we conducted two principal factor analyses to explore the relationships between the pet questions included in the child and PCG surveys and single measures of human-animal attachment. A single factor was extracted for both the child (6 item) and PCG (3 item) samples. For the child sample the eigenvalue was 1.95; the PCG sample eigenvalue was 1.28. For the pet attachment measures, we conducted correlation analyses and computed the Cronbach's coefficient alpha. Table 1 summarizes the questions and the results of these analyses, including the overall Cronbach's alpha. The alpha coefficients for the analyses were 0.7518 (child) and 0.7329 (PCG), suggesting that the two sets of items have acceptable internal consistency.

For the children, the mean scores for the combined pet Attachment measure was 2.9 (*SD* = 0.63); for the PCGs, 2.8 (*SD* = 0.78). Additional descriptive results are summarized in Table 2. While almost half (48.9%) of the child sample was female, nearly 80% of the PCGs were female, consistent with most PCGs being the mothers. In the CDS 2014, 63% of the older children and 61% of the PCGs reported having one or more pets. Approximately 44% of both children and PCGs reported a single pet in their household. Among the pet families, dogs (73.7% child, 78.3% PCG) were the most common pet, followed by cats (17.8% child, 34.1% PCG). Differences between these numbers may be attributable to question wording: In the CDS, the children were asked if they had a favorite pet and what it was; whereas the PCG asked specifically about different types of pets.

**Table 2:** Descriptive statistics.

**Measure**	**%/mean**	**SD**
**CHILD SAMPLE**		
Female	48.9	
Age	13.0	2.61
Only child in household	19.5	
Family size	4.6	1.46
One pet in household	43.6	
Dog	73.7	
Cat	17.8	
Attachment Score	2.9	0.63
**PRIMARY CAREGIVER SAMPLE**		
Female	79.4	
Age	37.6	9.08
One child in household	35.2	
Family size	4.0	1.32
One pet in household	43.6	
Dog	78.3	
Cat	34.1	
Attachment score	2.8	0.78

ANOVA was used to test the significance of relationships between several key measures (see Table 2) and attachment. One-way ANOVA was used to test for significance by gender, age, one child in house under 18, family size, one pet in household, dog in household, and cat in household. Girls (*M* = 2.99, *F* = 8.36, *p* < 0.004) and women (*M* = 2.84, *F* = 8.16, *p* < 0.004) had significantly higher levels of attachment than boys (*M* = 2.87) and men (*M* = 2.70). Age was not significantly associated with attachment for either the child or PCG samples. Only one child in the house under age 18 was significantly associated with higher attachment for both children (*M* = 3.04, *F* = 7.52, *p* < 0.006) and PCGs (*M* = 2.87, *F* = 5.04, *p* < 0.02) compared to children (*M* = 2.90) and PCGs (*M* = 2.78) in households with more than one child. Family size was significantly associated with pet attachment for both children (*F* = 5.13, *p* < 0.0001) and PCGs (*F* = 2.48, *p* < 0.008), with higher attachment among the larger families. Children (*M* = 2.87, *F* = 6.29, *p* < 0.01) and PCGs (*M* = 2.70, *F* = 27.74, *p* < 0.0001) who reported having only one pet had slightly lower mean of attachment than other pet owners (child *M* = 2.97, PCG *M* = 2.90). This finding may reflect the diversity of pets with some pets such as turtles or fish being less interactive. Having a dog was significantly associated with higher attachment for both children (*M* = 2.99, *F* = 23.46, *p* < 0.0001) and PCGs (*M* = 2.92, *F* = 117.30, *p* < 0.0001), with the mean attachment score higher for dog owners than for other pet owners (child *M* = 2.76, PCG *M* = 2.42). The results for cats were mixed: for children with cats the attachment scores were not significantly different from those without cats (but other pets); for the PCGs, cat ownership was significantly related to higher attachment (*M* = 2.95, *F* = 26.37, *p* < 0.0001) compared to other pet owners (*M* = 2.74).

## Discussion

This purpose of this paper was to describe and evaluate two shortened versions of the CENSHARE PAS that were incorporated into the PSID CDS 2014. Principal factor and correlation analyses of our 6-item older child and 3-item PCG versions of the PAS demonstrated a single factor, general attachment, and acceptable reliability. Using 29 items, Holcomb et al. (19) had identified 2 subscales within the original CENSHARE PAS: relationship maintenance (16 items) and intimacy (11 items). These two subscales had similar scores (3.16, 3.17) and were moderately correlated. Of the questions included in the CDS-2014, the exercise (child & PCG), moods (child), and greeting (child) questions were identified as parts of the Holcomb et al.'s (19) relationship maintenance subscale; the comfort (child & PCG), family (child & PCG), and study, read or watch TV (child) questions were identified as parts of their intimacy subscale.

Our findings of a single factor are due in part to the limited number of items included in the two measures. The larger instrument will likely continue to be useful for smaller studies, where researchers may be more focused on describing the dimensions of the human-animal bond. The lower scale scores (2.93, 2.81) for these brief scales may also reflect the limited nature of the shortened items. These issues reflect some of the limitations of the current analyses: The current findings may be missing some of the nuance of the multiple attachment subscales. In addition, the design of the parent study, the PSID CDS, focused on child development and well-being. This limited both the number of scale questions and additional pet-related questions. Nonetheless, we argue that the benefits outweigh the limitations and encourage other researchers to explore the use of shorter measures in large, ongoing studies to incorporate a general measure of human-animal attachment in studies that may focus on broader social, behavioral, and health topics.

In comparing some of the ANOVA results, several findings are consistent with those of the earlier study including: greater levels of attachment among girls and women and lower levels of attachment in larger households. We also find higher levels of attachment among dog owners. Findings for the cats are mixed with, no significant relationship in the child sample, but a positive relationship for the PCGs. This may reflect the greater relative reliance of the PCG short scale on the intimacy subscale (2 of 3 items) of the PAS. These differences underscore the need to measure the presence of pets, as well as attitudes toward pets, consistently both within and across surveys.

The field of human-animal interaction continues to grow and there is an increasing need for shorted, validated measures of dimensions of human-animal relationships. This paper has demonstrated multiple benefits of the development and use of brief attachment scales. Shorter scales can be cost-effective when seeking to include measures in large, population representative studies such as the PSID where space is at a premium. In the current paper, we have demonstrated that the shorter scales appear to be reliable and valid indicators of a general measure of pet attachment for older children and their primary caregivers. There is an ongoing need to explore the validity of brief measures in more detail and conduct more detailed analyses of differences in attachment across both human and pet characteristics and among additional populations, particularly younger children, where measurement of relational bonds via self-report measures can be challenging. Future work will take a deeper look at the current data to explore the relationship between pet attachment and multiple dimensions of family and child well-being and development.

## Ethics Statement

This article involves secondary analysis of publicly available data. It does not contain any studies with human participants or animals performed by any of the authors. Therefore, ethical approval was not required.

## Author Contributions

RB, MM, and NG all contributed to the abstract, literature review, introduction, and discussion. RB conducted the data analyses and drafted the data and methods and results sections.

### Conflict of Interest Statement

The authors declare that the research was conducted in the absence of any commercial or financial relationships that could be construed as a potential conflict of interest.
